# Balance design for robust foliar nutrient diagnosis of “Prata” banana (*Musa* spp.)

**DOI:** 10.1038/s41598-018-32328-y

**Published:** 2018-10-09

**Authors:** José Aridiano Lima de Deus, Júlio César Lima Neves, Márcio Cleber de Medeiros Corrêa, Serge-Étienne Parent, William Natale, Léon Etienne Parent

**Affiliations:** 1 Federal University of Ceará, Department of Soil Science, Fortaleza, 60440-554 Ceará Brazil; 2 Federal University of Viçosa, Department of Soils, Viçosa, 35670-900 Minas Gerais Brazil; 3 Federal University of Ceará, Department of Plant Science, Fortaleza, 60440-554 Ceará Brazil; 4 Université Laval, Department of Soils and Agri-Food Engineering, Québec, G1V 0A6 Québec, Canada

## Abstract

The “Cavendish” and “Prata” subgroups represent respectively 47% and 24% of the world banana production. Compared to world average progressing from 10.6 to 20.6 t ha^−1^ between 1961 and 2016, and despite sustained domestic demand and the introduction of new cultivars, banana yield in Brazil has stagnated around 14.5 t ha^−1^ mainly due to nutrient and water mismanagement. “Prata” is now the dominant subgroup in N-E Brazil and is fertigated at high costs. Nutrient balances computed as isometric log-ratios (*ilr*) provide a comprehensive understanding of nutrient relationships in the diagnostic leaf at high yield level by combining raw concentration data. Although the most appropriate method for multivariate analysis of compositional balances may be less efficient due to non-normal data distribution and limited nutrient mobility in the plant, robustness of the nutrient balance approach could be improved using Box-Cox exponents assigned to raw foliar concentrations. Our objective was to evaluate the accuracy of nutrient balances to diagnose fertigated “Prata” orchards. The dataset comprised 609 observations on fruit yields and leaf tissue compositions collected from 2010 to 2016 in Ceará state, N-E Brazil. Raw nutrient concentration ranges were ineffective as diagnostic tool due to considerable overlapping of concentration ranges for low- and high-yielding subpopulations at cutoff yield of 40 Mg ha^−1^. Nutrient concentrations were combined into isometric log-ratios (*ilr*) and normalized by Box-Cox corrections between 0 and 1 which may also account for restricted nutrient transfer from leaf to fruit. Despite reduced *ilr* skewness, Box-Cox coefficients did not improve model robustness measured as the accuracy of the Cate-Nelson partition between yield and the multivariate distance across *ilr* values. Sensitivity was 94%, indicating that low yields are attributable primarily to nutrient imbalance. There were 148 false-positive specimens (high yield despite nutrient imbalance) likely due to suboptimal nutrition, contamination, or luxury consumption. The profitability of “Prata” orchards could be enhanced by rebalancing nutrients using *ilr* standards with no need for Box-Cox correction.

## Introduction

The “Cavendish” and “Prata” subgroups represent 47% and 24% of the world banana production, respectively^1^. The current cultivars of bananas originate from the hybridization of diploid subspecies of *Musa acuminata* Colla (A genome) and *Musa balbisiana* Colla (B genome), and they exhibit various levels of ploidy and genomic constitution, such as diploid (AA; BB; or AB; 2n = 2x = 22); triploid (AAA; AAB; or ABB; 2n = 3x = 33); and tetraploid (AAAA; AAAB; AABB; or ABBB; 2n = 4x = 44)^2^. The main banana cultivars in Brazil have genomes AA, AAA, AAB, ABB, and AAAB^2^. Brazil produces 6.9 × 10^6^ tons of banana fruits annually on 480 × 10^3^ hectares^3^. Although banana production in Brazil is supported by strong domestic demand representing 98% of total production^4^, the average yield of 14.5 tons ha^−1^ year^−1^ remains lower than the world average^5^ of 20.6 tons ha^−1^ year^−1^.

Banana nutrient requirements depend on yield potential, plant density, soil fertility, and root development^6^. Low fruit yields are generally attributed to nutrient mismanagement and water shortage^7–10^. The K is generally the main limiting nutrient and interacts with N, Ca and Mg^11^. Because K is also of public health concern due to too low daily intake^12–15^, it is applied in relatively large amounts to boost banana yield and quality, potentially leading to luxury consumption. “Prata” is the dominant banana subgroup in N-E Brazil and is fertigated. Fertilization represents 16–22% of production costs compared to 14–27% for irrigation^16^.

Nutrient acquisition by the banana fruit relies on plant’s ability to translocate nutrients. N, P, K, Mg, Cu, and Zn have the greatest potential for nutrient translocation from leaf to fruit^17–19^. S, Cu, Zn, Mn and Fe have variable mobility, and Ca and B are relatively immobile^20,21^. The indirect relationship between relatively phloem-immobile leaf nutrients and fruit yield depends on soil test and water supply regulating xylem transport to the fruit^22^. As source of variability and heteroscedasticity nutrient mobility from leaf to fruit could be constrained by Box-Cox exponents^23^ between zero (immobile nutrients unrelated to yield) and 1 (mobile nutrients related to yield).

Banana diagnostic leaf tissue is collected close to blooming stage^11^. There are several methods to interpret the results of tissue analysis. The most common method is the critical concentration range diagnosing nutrients separately^24^, assuming that all other factors are close to their optima^25^, an over-optimistic assumption. Because components of a system are intrinsically interactive and multivariate^26^, nutrients can be combined into balances using isometric log-ratios (*ilr*)^27^. The *ilr* transformation is the most suitable method to run multivariate analyses on compositional data^28^ such as leaf composition^29–31^. However, the *ilr* transformation may not return normality or homoscedasticity.

Box-Cox coefficients assigned to raw concentration data could improve *ilr* data distribution and model accuracy. Exponents may be varied between 0 and 1, zero not contributing to *ilr* (${c}^{0}=1$) and 1, fully contributing to it (${c}^{1}=c$); intermediate Box-Cox coefficients reflect partial contribution. Model accuracy is commonly determined after partitioning data into true-negative (TN), false-negative (FN), true-positive (TP) and false-positive (FP) specimens using the Cate-Nelson procedure, the receiving operating characteristic (ROC) or the confusion matrix^29,30^. Accuracy is computed as (TN+TP)/(TN+FN+TP+FP). However, nutrient excess at high yield level (FP specimens) is diagnosed as sub-optimal concentration, luxury consumption of contamination. Accuracy defined in terms of diagnostic power could thus be computed as (TN+TP+FP)/(TN+FN+TP+FP). The conventional *ilr* transformation is robust if Box-Cox coefficients of one across concentrations return greatest model accuracy and diagnostic power after varying coefficients between zero and one.

Our objective was to measure the robustness, accuracy and diagnostic power of nutrient balance designs for fertigated “Prata” banana. We hypothesized that high yields of fertigated “Prata” are reached within narrow foliar nutrient combinations into balances after optimizing Box-Cox coefficients assigned to raw concentration data.

## Results

### Yield partitioning and Box-Cox transformation

**Figure 1:**
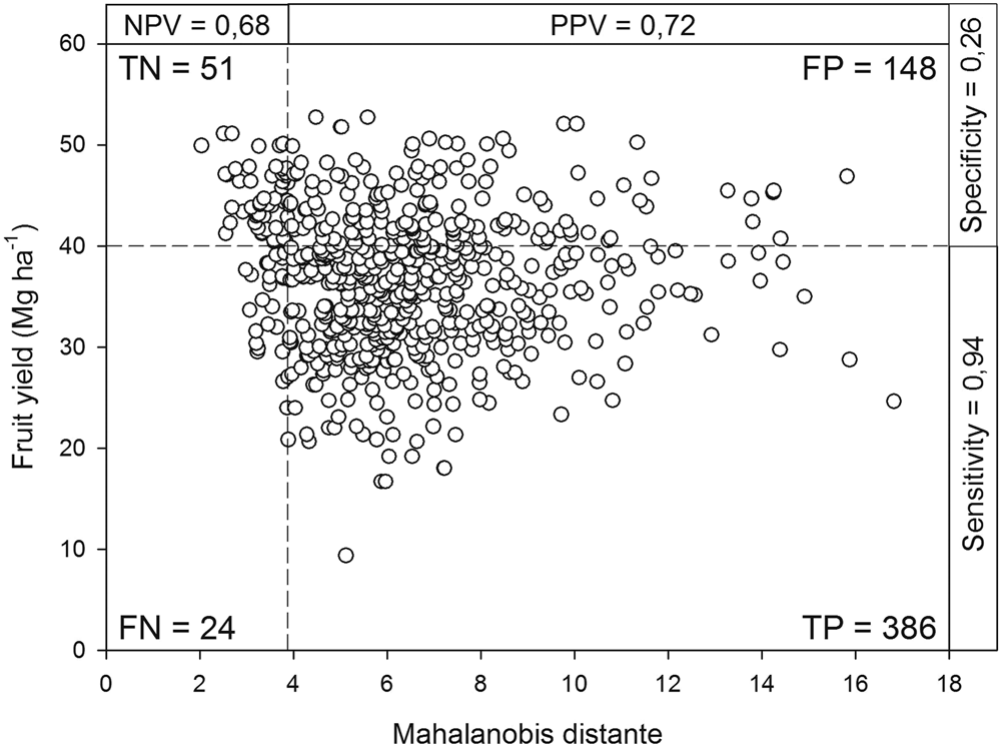
Cate–Nelson partitioning of the relationship between Mahalanobis distance and banana yield (critical distance = 3.9 at cutoff yield of 40 Mg ha^−1^). TN = true-negative, FN = false-negative, TP = true-positive, FP = false-positive.

Due to irrigation, fruit yield did not differ significantly between the wet (18.35 ± 4.39 Mg ha^−1^) and dry (18.35 ± 3.77 Mg ha^−1^) seasons. Fruit yields were normally distributed in both seasons and varied between 9 and 53 tons ha^−1^ year^−1^. The critical Mahalanobis distance across the whole dataset was 3.9 at a cutoff yield of 40 Mg fruit ha^−1^ (Fig. 1). The partition showed 8.4% TN, 3.9% FN, 63.4% TP, and 24.3% FP specimens. Combining nutrient concentrations into *ilr* values returned an accuracy of 72%, negative predictive value of 68%, positive predictive value of 72%, and sensitivity of 94%. The diagnostic power was 96%. Specificity was 26% due to the high number of FP specimens. The *ilr* of the TN subgroup (*n* = 51 specimens) used to compute the Mahalanobis distance are presented as (Supplementary Material Table 1). Using the traditional critical value approach, it was not possible to establish “adequate” nutrient concentration ranges due to considerable overlap between high- and low-yielding subpopulations (Table 1). Imbalanced nutrition was the main cause of low yields in these intensively managed production systems. The proportion of imbalanced crops, determined as (FP+TP)/total, was 88%. Thus, fertilization regimes were suboptimal in most orchards.

**Table 1:** Ranges of concentration values for two yield classes.

Nutrient	Minimum	Median	Maximum	Minimum	Median	Maximum
	High yield ≥ 40 tons ha^−1^			Low yield < 40 tons ha^−1^		
	g kg^−1^					
N	18.2	21.7	26.8	16.4	21.9	27.0
P	1.0	1.7	2.9	0.7	1.6	2.7
K	13.8	33.5	59.5	14.4	33.5	48.6
Ca	2.9	6.5	11.1	2.6	6.3	10.3
Mg	1.6	2.4	3.9	0.8	2.4	4.1
S	1.0	1.5	2.2	0.7	1.5	2.0
	mg kg^−1^					
B	3	11	21	1	10	11
Cu	3	6	13	2	5	21
Fe	41	65	105	29	66	100
Mn	22	175	403	18	132	539
Zn	8	16	26	7	16	32
Na	10	44	100	10	48	100
Al	2	27	77	2	28	80

## Discussion

Although nutrient imbalance appeared to be the main yield-limiting factor in Ceará state, there were 24 FN specimens in which yields were limited by other factors. There are several classes of soil that enable the soil to sustain banana production relative to soil properties^32^. High-yielding crops in Group 1 were grown in level or gently undulating well-drained soils with solum thickness exceeding 100 cm and with loam to clayey texture. Group 1 soils were well-structured, fertile, and neutral to slightly acidic, with no saline limitations. Medium-yielding crops in Group 2 were limited by natural fertility, slope, solum thickness, or drainage, and required investment to reach high yield. Low-yielding crops in Group 3 soils were limited by low soil fertility level, sandy texture, or solum thickness (30–35 to 75 cm). Very low-yielding crops in Group 4 soils had several major limitations, such as shallow (<25 cm) solum and high salinity. Soil bulk density, pH, and electrical conductivity did not limit yield in Ceará^33^, leaving solum thickness as the probable yield-limiting factor. Quartzispamments require major investments in fertilization and irrigation due to low nutrient and water reserves in Ceará soils (Group 3).

Levels of banana leaf nutrients such as Ca, Mg, and Mn were relatively stable at daytime temperatures between 17 and 30 °C^34^, such as those typical of Missão Velha. Mg did not appear to be yield-limiting at Missão Velha despite high K levels that are potentially antagonistic to Mg^6,17,18^. However, Ca, B, and S may accumulate as a result of local soil properties, such as high pH and organic matter content^35^. The S level likely reflected water quality due to the presence of gypsum layers in the sedimentary basin at Missão Velha^36^. The relative shortage of Mn can be generally attributed to high soil pH^37^. Banana plants are highly responsive to added Mn^38^, but foliar Mn diagnostic standards are elusive^9^. Although often shown to be in relative shortage in banana orchards^9,39^, Zn levels were adequate or in apparent excess at Missão Velha. The [B | Ca] balance among TP specimens was often high, which indicated potential Ca excess or B shortage.

The status of Na and Al in banana leaf has been little documented. Accumulation of Na in banana seedlings may reduce biomass production^33,40^. Exponential increase from 0.6 to 3.1 g Na kg^−1^ was observed in the shoots of banana seedlings^33^, well below the Na level of 25 g kg^−1^ in leaf margins suffering from necrosis^40^. Any beneficial role of Na could occur below around 1 g Na kg^−1^, as is the case in Missão Velha. Foliar Al concentration^41^ normally ranges between 0.050 and 0.400 g Al kg^−1^. Foliar Al may accumulate in high-pH soils due to the attack of soil-borne pathogens on banana roots^42^. Roots react by exuding oxalic, malic, and fumaric acids^43^, which may chelate Al ions^44,45^. As a result, tissue Al varied widely between orchards but did not reach toxic levels.

The cutoff yield to establish nutrient standards was 40 tons ha^−1^. For AAB banana a cutoff yield of 30 tons ha^−1^ was suggested in Minas Gerais, Brazil^46^, while for AAA banana a cutoff yield close to 40 tons ha^−1^ was stipulated in São Paulo state^47^. In Ceará state, commercial yields of AAB “Prata” are generally classified as low (<30 tons ha^−1^), medium (30–35 tons ha^−1^), or high (≥35 tons ha^−1^). In the Ceará dataset, 106 orchards produced <30 tons ha^−1^, 137 orchards produced 30–35 tons ha^−1^, 317 orchards produced 35–45 tons ha^−1^, and 49 orchards produced >45 tons ha^−1^. Furthermore, 199 orchards (33%) produced ≥40 tons ha^−1^. Obviously, there is a large potential to increase yield in Ceará orchards by rebalancing nutrients.

Whereas nutrients diagnosed separately may return low diagnostic performance^31^, ternary diagrams and multivariate analyses allow the entire nutrient status to be captured^48,49^. Additionally, criticism of the misuse of multivariate analysis to analyze compositional data is reported in the literature^26^. Log-ratio transformations address resonance in the compositional space (if one concentration increases, one or more concentration values must decrease), subcompositional incoherence, and the intrinsically non-normal distribution in the constrained compositional space compared to the unconstrained real space required to conduct statistical analyses. The *clr* transformation^50^ is the most common log-ratio used to diagnose nutrient limitations in crops. To facilitate interpretation of the diagnosis from the Mahalanobis distance, *clr* indices can be ordered in ascending order from the most negative (relative shortage) to the most positive (relative excess), as in DRIS^51^.

The diagnostic accuracy of 72% reported here was lower than the generally obtained value of 80%, whereas the critical Mahalanobis distance of 3.9 was close to the range of 4.1–5.9 that has been reported for other crops^29,31,52,53^. Low accuracy was due to the high proportion of FP specimens (24%), indicating suboptimal fertilization, luxury consumption of nutrients, or leaf contamination. Fertigation appeared to be adequately run in 12% of orchards (TN+FN). Pests or unfavorable soil factors may have limited the yield of FN specimens. The fertigation regime should be rebalanced in 88% of orchards (FP+TP). Thus, nutrient mismanagement appeared to be the main yield-limiting factor at Missão Velha. Regional nutrient standards appeared to be more reliable than so-called “universal” nutrient ratio standards^35^.

## Conclusion

Nutrient imbalance limited yield in Ceará’s “Prata” banana orchards. Nutrient concentration ranges did not discriminate between low- and high-yielding crops but combining nutrients into *ilr* and *clr* made possible performing nutrient standards. The Cate–Nelson partitioning about cutoff yield of 40 tons ha^−1^ and the multivariate distance across *ilr* values returned a critical Mahalanobis distance of 3.9, below which nutrient balances were adequate. Although appealing to improve data distribution and constrain nutrient mobility in plants, the Box-Cox coefficients assigned to raw nutrient concentrations failed to improve model accuracy.

The order of nutrient limitation to yield shown by *clr* indices should be further validated by using nutrient tests for specimens showing the Mahalanobis distance above the critical value. While 4% of orchards (FN specimens), solum thickness or other factors limited fruit yield, 96% of orchards were classified as nutritionally balanced (TN specimens) or imbalanced (FP and TP specimens). The most commonly deficient nutrient was boron, and the most commonly excessive was nitrogen. Although fertigation can enhance banana yield, the dosage of nutrients requires adjustment based on reliable diagnostic tools. Banana is the fruit crop showing yet the highest proportion of false positive specimens and the smallest number of false negative specimens. Hence nutrient imbalance is a crucial problem that could be addressed using tools of compositional data analysis.

## Methods

### Climatic and soil conditions

Data were collected from 2010 to 2016 in 6- to 18-year-old banana stands at Missão Velha, Ceará state, Brazil (7° 35′ S and 39° 21′ W, 442 m in altitude). Climate in this part of Brazil is semi-arid tropical (Aw in the Köppen-Geiger classification), with a dry winter season and rainfall concentrated in the summer season. The warmest months in the area extend from September to December (Supplementary Table 2)^54^. Maximum and minimum temperature ranges were 31–35 °C and 19–21 °C, respectively. For comparison, optimal mean temperatures for banana crops are as follows: 22 °C for floral initiation, 31 °C for leaf growth and development, and 28 °C (range: 15–35 °C) for high commercial yields^32^. Total rainfall averaged 1022.6 mm, compared to 1200–1800 mm as an effective precipitation regime for banana production^32^.

Soils are sandy and classified as Neossolo Quartzarênico^55^ or Quartzipsamment^56^. Soil properties in the top layer (0–20 cm) are presented as (Supplementary Table 3).

### Orchard management

Commercial orchards averaging 3 ha in size were surveyed for yield and foliar composition. Plant density averaged 1332 plants ha^−1^ (4.0 m × 2.0 m double row, 2.5 m between plants). Plants were sprinkler-irrigated at a crop evapotranspiration (*ET*_*c*_) rate proportional to potential evapotranspiration *ET*_0_ (*ET*_*c*_ = *K*_*C*_*ET*_0_, where *K*_*C*_ varied during the season)^6,57^. To sustain high yield and quality, the banana clump was restricted to a few fruiting plants by yearly pruning. Banana yields could be affected not only by nutrient imbalance but also by diseases and pests, including nematodes^58^. The most frequent pest is the banana weevil (*Cosmopolites sordidus*), and the most frequent disease is yellow Sigatoka (*Mycosphaerella musicola*).

In fertigated orchards, nitrogen is generally supplied as urea (45% N) every 3 to 15 days up to 440–600 kg N ha^−1^ year^−1^, as follows: 10% during the first 3 months, 75% between the fourth month and blooming (seventh to ninth month), and 15% up to harvest. Depending on the soil test, potassium is applied as potassium chloride (60% of K_2_O) every 3–15 days at total rate of 1300 to 1700 kg K_2_O ha^−1^ year^−1^ as follows: 0% during the first 3 months, 90% from the fourth month to blooming, and 10% toward harvest. Phosphorus is applied as reactive natural phosphate (27% of P_2_O_5_) at planting and via fertigation as monoammonium phosphate (48% of P_2_O_5_) at total rate of 160 kg P_2_O_5_ ha^−1^ year^−1^. Calcium is supplied to maintain the soil K: Ca: Mg molar ratio between 0.5: 3.5: 1.0 and 0.3: 2.0: 1.0. Manganese, zinc, and boron are applied at rates of 60 kg manganese sulfate (26% Mn) ha^−1^ year^−1^, 96 kg zinc sulfate (20% Zn) ha^−1^ year^−1^, and 20–30 kg boric acid (17% B) ha^−1^ year^−1^, respectively.

### Data collection and tissue analysis

The dataset comprised 609 observations of cv. ‘Prata’, AAB “Prata” subgroup. The period between budding and harvest varied from 8 to 12 months. Yield data were reported for the dry and wet seasons. In July and December of each year, the third most fully expanded leaf of banana plants was collected at blooming stage twice a year during the wet and dry seasons, and 10-cm-wide pieces were cut from inner halves on both sides of the midrib and at the midpoint of the lamina^59^. Four samples made of 10 subsamples were composited in each orchard. Samples were oven-dried at 72 °C for 48–96 hours and ground to less than 1 mm. N was determined by the micro-Kjeldahl method. After sample digestion in a mixture of nitric and perchloric acids^60^, the elements Ca, Mg, Fe Zn, Cu, Al and Mn were quantified by atomic absorption spectrophotometry, P by colorimetry, S by turbidimetry, and K and Na by emission flame photometry^61^. B was quantified by colorimetry^62^.

### Log-ratio and Box-Cox transformations

The tissue compositional simplex comprised N, P, K, Ca, Mg, S, Cu, Zn, Mn, Fe, B, Na, and Al, as well as a filling value computed as the difference between the measurement unit and the sum of nutrients. The orthonormal balance was computed as the *ilr*, as follows^63,64^:

$$il{r}_{j}=\sqrt{\frac{{n}_{j}^{+}{n}_{j}^{-}}{{n}_{j}^{+}+{n}_{j}^{-}}}ln\frac{g({c}_{numerator}^{{\rm{p}}})}{g({c}_{denominator}^{{\rm{q}}})},$$

where *n*_*j*_^+^ and *n*_*j*_^−^ are numbers of components at the numerator and denominator, respectively, $g({c}_{numerator}^{{\rm{p}}})$ and $g({c}_{denominator}^{{\rm{q}}})$ are geometric means across components at the numerator and the denominator, respectively, p and q are Box-Cox coefficients assigned to components at numerator and denominator, respectively, and $\sqrt{{n}_{j}^{+}{n}_{j}^{-}/({n}_{j}^{+}+{n}_{j}^{-})}$ is a normalization coefficient. The *ilr* is designated as [components at denominator|components at numerator] because the log-ratio becomes more negative as values at the denominator increase. More negative numbers are located on the left-hand side of the array, as in algebra. The *ilr* values were used to compute the Mahalanobis distance from a reference subpopulation^65^. Box-Cox coefficients varying between 0 and 1 are assigned to raw concentrations at numerator and denominator, hence transforming *ilr* values to reach additivity, normality or homoscedasticity and possibly controlling nutrient mobility, a coefficient near zero making the nutrient immobile and a coefficient close to one making the nutrient mobile.

The balance design was elaborated following a sequential binary partition (Supplementary Table 4). N, P, K, and Mg are mobile nutrients, S, Cu, Zn, Mn, and Fe are of variable mobility, and Ca and B are relatively immobile^20,21^. K and Mg are antagonistic to each other^22^. N and P reflect protein synthesis and energy transport, respectively^66^. Among nutrients of limited mobility, S is involved in protein synthesis, whereas Cu, Zn, and Mn are involved in metabolism^22^ and fungicide formulations. Fe and Mn are involved in soil genesis^55^. Sodium (Na) acts as a functional element as osmoticium for cell enlargement and an accompanying cation for long-distance transport^67^. Plant tolerance to Al toxicity depends on Al interactions with other minerals, such as B, P, Ca, and Mg^41^.

### Statistical analysis

Computations were performed by using the R statistical package version 3.4.1^68^. The dataset was separated into calibration (2010–2014) and validation (2015–2016) datasets that were merged in case of similar accuracy. The Cate–Nelson procedure partitions data into true-negative (TN), false-negative (FN), true-positive (TP), and false-positive (FP) quadrants from the relationship between crop yield and Mahalanobis distance^65^. Specificity is computed as TN/(TN+FP), sensitivity as TP/(TP+FN), accuracy as (TN+TP)(TN+FN+TP+FP), negative predictive value as TN/(TN+FN), and positive predictive value as TP/(TP+FP). The centered log-ratio (*clr*) values of the TN subpopulation were compared with published data after adjusting the geometric mean for the number of components (Table 2).

**Table 2:** Comparison of centered log-ratio standards (mean and standard deviation [SD]) for the banana diagnostic leaf in the present study (6–14 components), India (10 components), and Uganda (6 components).

*clr*	Ceará standards			India^‡^	Uganda^§^
	Mean ± SD (14)	Mean ± SD (10)	Mean ± SD (6)	Mean ± SD (10)	Mean ± SD (6)
V_N_	3.670 ± 0.098	2.259 ± 0.102	0.315 ± 0.065	2.226 ± 0.199 ns	0.302 ± 0.153 ns
V_P_	1.073 ± 0.100	−0.339 ± 0.100	−2.282 ± 0.076	−0.505 ± 0.289**	−2.312 ± 0.176 ns
V_K_	4.159 ± 0.117	2.747 ± 0.119	0.804 ± 0.102	2.485 ± 0.231**	0.607 ± 0.168**
V_Mg_	1.396 ± 0.116	−0.015 ± 0.115	−1.959 ± 0.093	0.318 ± 0.449**	−1.546 ± 0.199**
V_S_	0.997 ± 0.076	−0.415 ± 0.076	—	−1.057 ± 0.361**	na
V_Cu_	−4.632 ± 0.109	—	—	na	na
V_Zn_	−3.613 ± 0.100	−5.024 ± 0.095	—	−4.815 ± 0.359**	na
V_Mn_	−1.208 ± 0.525	−2.619 ± 0.515	—	−2.246 ± 0.412**	na
V_Fe_	−2.191 ± 0.093	−3.602 ± 0.098	—	−3.212 ± 0.384**	na
V_Ca_	2.412 ± 0.096	1.001 ± 0.093	−0.943 ± 0.098	1.121 ± 0.432 ns	−0.859 ± 0.289 ns
V_B_	−3.834 ± 0.291	—	—	na	na
V_Na_	−2.451 ± 0.361	—	—	na	na
V_Al_	−3.196 ± 0.225	—	—	na	na
V_Fv_^†^	7.419 ± 0.095	6.007 ± 0.092	4.066 ± 0.058	5.685 ± na**	3.809 ± 0.087**

Note: ^†^Fv, filling value; ‡^9^; §^10^; na, not available; ns, not significantly different from Ceará standards according to two-tailed *t*-test; **P = 0.01 vs. Ceará standards according to two-tailed *t*-test.

## Electronic supplementary material

SUPPLEMENTARY MATERIAL
