# Supplementary material for: Balance design for robust foliar nutrient diagnosis of “Prata” banana (Musa spp.)
Source: Sci Rep. 2018 Oct 9;8:15040. doi: 10.1038/s41598-018-32328-y (PMC6177482; doi:10.1038/s41598-018-32328-y)
Supplement: Supplementary file 1 — SUPPLEMENTARY MATERIAL [file 41598_2018_32328_MOESM1_ESM.docx]

**Balance design for robust foliar nutrient diagnosis of “Prata” banana (*Musa* spp.)**

José Aridiano Lima de Deus^1*^, Júlio César Lima Neves^2^, Márcio Cleber de Medeiros Corrêa^3^, Serge-Étienne Parent^4^, William Natale^3^, and Léon E. Parent^4^

^1^Federal University of Ceará, Department of Soil Science, Fortaleza, 60440-554, Ceará, Brazil.

^2^Federal University of Viçosa, Department of Soils, Viçosa, 35670-900, Minas Gerais, Brazil.

^3^Federal University of Ceará, Department of Plant Science, Fortaleza, 60440-554, Ceará, Brazil.

^4^Université Laval, Department of Soils and Agri-Food Engineering, Québec, G1V0A6, Québec, Canada.

*Corresponding author: [aridianolima@yahoo.com.br](mailto:aridianolima@yahoo.com.br)

## **SUPPLEMENTARY MATERIAL**

| *ilr* | *ilr*1 | *ilr*2 | *ilr*3 | *ilr*4 | *ilr*5 | *ilr*6 | *ilr*7 | *ilr*8 | *ilr*9 | *ilr*10 | *ilr*11 | *ilr*12 | *ilr*13 |
| --- | --- | --- | --- | --- | --- | --- | --- | --- | --- | --- | --- | --- | --- |
|  | Inverse covariance matrix | | | | | | | | | | | | |
| *ilr*1 | 288.113 | -43.632 | -1.995 | -91.160 | 98.952 | -11.409 | -45.295 | -5.257 | 44.840 | 1.327 | 12.428 | 41.604 | 158.250 |
| *ilr*2 | -43.632 | 135.703 | 12.531 | -25.592 | 59.916 | -4.594 | 32.920 | 19.770 | -48.180 | 33.604 | 16.042 | -37.499 | -31.244 |
| *ilr*3 | -1.995 | 12.531 | 386.830 | 226.270 | -174.188 | -12.428 | 14.089 | -38.857 | -13.224 | 35.911 | 25.115 | -13.345 | 123.389 |
| *ilr*4 | -91.160 | -25.592 | 226.270 | 428.980 | -451.591 | 29.014 | 46.290 | -7.122 | 23.945 | 11.035 | 4.976 | 0.136 | -14.506 |
| *ilr*5 | 98.952 | 59.916 | -174.188 | -451.591 | 746.365 | -63.413 | 15.211 | -4.506 | -16.925 | -2.789 | 16.585 | -14.534 | 172.243 |
| *ilr*6 | -11.409 | -4.594 | -12.428 | 29.014 | -63.413 | 155.478 | -16.874 | -19.461 | -39.508 | 36.362 | 12.809 | -0.746 | -50.429 |
| *ilr*7 | -45.295 | 32.920 | 14.089 | 46.290 | 15.211 | -16.874 | 75.063 | 52.069 | -18.701 | 8.313 | 1.131 | -20.412 | -16.637 |
| *ilr*8 | -5.257 | 19.770 | -38.857 | -7.122 | -4.506 | -19.461 | 52.069 | 81.800 | -10.058 | 5.773 | -2.273 | -5.748 | -10.946 |
| *ilr*9 | 44.840 | -48.180 | -13.224 | 23.945 | -16.925 | -39.508 | -18.701 | -10.058 | 123.549 | -65.645 | -4.519 | 13.753 | 37.072 |
| *ilr*10 | 1.327 | 33.604 | 35.911 | 11.035 | -2.789 | 36.362 | 8.313 | 5.773 | -65.645 | 65.922 | 15.963 | 0.616 | 14.968 |
| *ilr*11 | 12.428 | 16.042 | 25.115 | 4.976 | 16.585 | 12.809 | 1.131 | -2.273 | -4.519 | 15.963 | 17.459 | -4.271 | 30.130 |
| *ilr*12 | 41.604 | -37.499 | -13.345 | 0.136 | -14.534 | -0.746 | -20.412 | -5.748 | 13.753 | 0.616 | -4.271 | 41.427 | 48.576 |
| *ilr*13 | 158.250 | -31.244 | 123.389 | -14.506 | 172.243 | -50.429 | -16.637 | -10.946 | 37.072 | 14.968 | 30.130 | 48.576 | 404.618 |
|  | Mean | | | | | | | | | | | | |
|  | 1.834 | 1.940 | -0.412 | 6.997 | 3.485 | -0.737 | 0.708 | -2.442 | 0.881 | 4.471 | 2.225 | 2.733 | -7.696 |

**Supplementary Table 1.** Inverse covariance matrix and mean of *ilr* values of TN specimens (*ilr*1-13 defined according to the sequential binary partition in Supplementary Table 4).

| Month | Precipitation | NDP | T_max_ | T_min_ | RH | Insolation |
| --- | --- | --- | --- | --- | --- | --- |
|  | mm | - | °C | | % | hours |
| January | 177.8 | 13 | 32.9 | 21.1 | 74.6 | 215 |
| February | 159.6 | 13 | 32.5 | 21.0 | 82.2 | 203 |
| March | 269.6 | 14 | 32.3 | 21.3 | 82.2 | 240 |
| April | 154.4 | 11 | 31.9 | 21.0 | 82.6 | 239 |
| May | 70.5 | 6 | 32.0 | 20.6 | 76.5 | 237 |
| June | 37.8 | 4 | 31.5 | 19.6 | 72.6 | 231 |
| July | 10.2 | 2 | 31.7 | 19.3 | 67.2 | 244 |
| August | 1.5 | 1 | 33.2 | 19.0 | 59.3 | 290 |
| September | 0.9 | 0 | 34.7 | 19.8 | 54.8 | 296 |
| October | 39.4 | 4 | 35.4 | 21.2 | 56.4 | 285 |
| November | 30.4 | 5 | 34.9 | 21.7 | 60.4 | 260 |
| December | 70.5 | 8 | 34.2 | 21.7 | 64.8 | 245 |
| Total |  | 81 | - | - | - | 2985 |
| **Supplementary Table 2.** Average monthly climatic data for Missão Velha, Ceará state, Brazil, from 2010 to 2016, INMET^68^. Note: NDP = number of days with precipitation; T_max_ = maximum temperature; T_min_ = minimum temperature; RH = relative humidity. | | | | | | |

##

| Property | Minimum | Median | Maximum |
| --- | --- | --- | --- |
| pH_CaCl2_ | 6.3 | 7.2 | 7.9 |
| Organic matter (*g dm^-3^*) | 5.0 | 21.8 | 39.0 |
| Mehlich-1 P (*mg dm^-3^*) | 15.0 | 119.8 | 300.0 |
| Exchangeable K (*mmol_c_ dm^-3^*) | 1.1 | 4.5 | 15.0 |
| Exchangeable Ca (*mmol_c_ dm^-3^*) | 15.0 | 58.2 | 184.5 |
| Exchangeable Mg (*mmol_c_ dm^-3^*) | 3.0 | 14.6 | 51.0 |
| Sum of cationic bases (*mmol_c_ dm^-3^*) | 22.2 | 77.4 | 225.7 |
| Cation exchange capacity (CEC) (*mmol_c_ dm^-3^*) | 34.2 | 89.4 | 233.8 |
| Base saturation (%) | 62.8 | 84.2 | 97.8 |

**Supplementary Table 3.** Soil analysis of Missão Velha, Ceará state, Brazil, from 2010 to 2016. Note: Soil samples (0–20 cm) composited at each site into 250-cm^3^ samples were air-dried, ground, and sieved to <2 mm for chemical analysis^61^. The pH was measured in 1:2.5 soil-to-water volumetric ratio. The P, K, Cu, Mn, Fe, and Zn were extracted by the Mehlich-1 method. The Ca, Mg, and Al were extracted with 1N KCl. The S was extracted with monocalcium phosphate (500 mg P L^-1^) diluted into 2M acetic acid in a 1:2.5 soil-to-solution ratio. Elements except B were quantified by inductively coupled plasma (ICP-OES). The B was extracted by the hot-water method and quantified by colorimetry. Exchangeable acidity (H + Al) was extracted using calcium acetate 0.5 M at pH 7.0. Total C was determined by dichromate oxidation (Walkley–Black) and multiplied by 1.724 to obtain organic matter content.

| *ilr* | N | P | K | Mg | S | Cu | Zn | Mn | Fe | Ca | B | Na | Al | F_v_ |
| --- | --- | --- | --- | --- | --- | --- | --- | --- | --- | --- | --- | --- | --- | --- |
| 1 | 1 | -1 | 0 | 0 | 0 | 0 | 0 | 0 | 0 | 0 | 0 | 0 | 0 | 0 |
| 2 | 0 | 0 | 1 | -1 | 0 | 0 | 0 | 0 | 0 | 0 | 0 | 0 | 0 | 0 |
| 3 | 1 | 1 | -1 | -1 | 0 | 0 | 0 | 0 | 0 | 0 | 0 | 0 | 0 | 0 |
| 4 | 1 | 1 | 1 | 1 | -1 | -1 | -1 | -1 | -1 | 0 | 0 | 0 | 0 | 0 |
| 5 | 0 | 0 | 0 | 0 | 1 | -1 | -1 | -1 | -1 | 0 | 0 | 0 | 0 | 0 |
| 6 | 0 | 0 | 0 | 0 | 0 | 1 | -1 | 0 | 0 | 0 | 0 | 0 | 0 | 0 |
| 7 | 0 | 0 | 0 | 0 | 0 | 0 | 0 | 1 | -1 | 0 | 0 | 0 | 0 | 0 |
| 8 | 0 | 0 | 0 | 0 | 0 | 1 | 1 | -1 | -1 | 0 | 0 | 0 | 0 | 0 |
| 9 | 1 | 1 | 1 | 1 | 1 | 1 | 1 | 1 | 1 | -1 | -1 | 0 | 0 | 0 |
| 10 | 0 | 0 | 0 | 0 | 0 | 0 | 0 | 0 | 0 | 1 | -1 | 0 | 0 | 0 |
| 11 | 1 | 1 | 1 | 1 | 1 | 1 | 1 | 1 | 1 | 1 | 1 | -1 | 0 | 0 |
| 12 | 1 | 1 | 1 | 1 | 1 | 1 | 1 | 1 | 1 | 1 | 1 | 1 | -1 | 0 |
| 13 | 1 | 1 | 1 | 1 | 1 | 1 | 1 | 1 | 1 | 1 | 1 | 1 | 1 | -1 |

**Supplementary Table 4.** Sequential binary partition of the banana leaf components, including the filling value (F_v_) between the sum of analytical results and the unit of measurement.
